# Chiari malformation type I and basilar invagination originating from atlantoaxial instability: a literature review and critical analysis

**DOI:** 10.1007/s00701-020-04429-z

**Published:** 2020-06-06

**Authors:** Arthur Wagner, Lukas Grassner, Nikolaus Kögl, Sebastian Hartmann, Claudius Thomé, Maria Wostrack, Bernhard Meyer

**Affiliations:** 1 Department of Neurosurgery, Klinikum rechts der Isar, Technical University Munich School of Medicine, Ismaninger Str. 22, 81675 Munich, Germany; 2 Department of Neurosurgery, Medical University Innsbruck, Innsbruck, Austria

**Keywords:** Atlantoaxial fusion, Atlantoaxial instability, Basilar invagination, C1-2 fusion, Chiari malformation, Syringomyelia

## Abstract

**Introduction:**

Recently, a novel hypothesis has been proposed concerning the origin of craniovertebral junction (CVJ) abnormalities. Commonly found in patients with these entities, atlantoaxial instability has been suspected to cause both Chiari malformation type I and basilar invagination, which renders the tried and tested surgical decompression strategy ineffective. In turn, C1-2 fusion is proposed as a single solution for all CVJ abnormalities, and a revised definition of atlantoaxial instability sees patients both with and without radiographic evidence of instability undergo fusion, instead relying on the intraoperative assessment of the atlantoaxial joints to confirm instability.

**Methods:**

The authors conducted a comprehensive narrative review of literature and evidence covering this recently emerged hypothesis. The proposed pathomechanisms are discussed and contextualized with published literature.

**Conclusion:**

The existing evidence is evaluated for supporting or opposing sole posterior C1-2 fusion in patients with CVJ abnormalities and compared with reported outcomes for conventional surgical strategies such as posterior fossa decompression, occipitocervical fusion, and anterior decompression. At present, there is insufficient evidence supporting the hypothesis of atlantoaxial instability being the common progenitor for CVJ abnormalities. Abolishing tried and tested surgical procedures in favor of a single universal approach would thus be unwarranted.

## Introduction

**Fig. 1:**
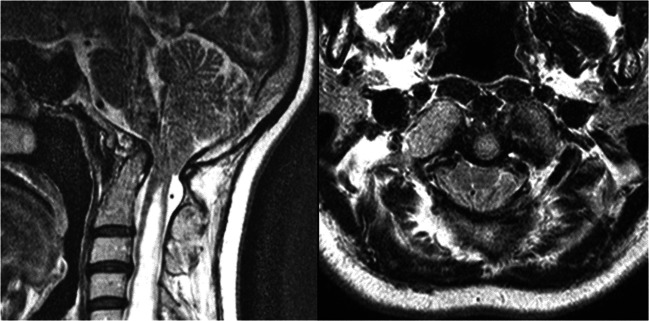
Chiari malformation type I with syringomyelia in a 46-year-old female. Magnetic resonance imaging, sagittal (**a**) and axial (**b**) planes

**Fig. 2:**
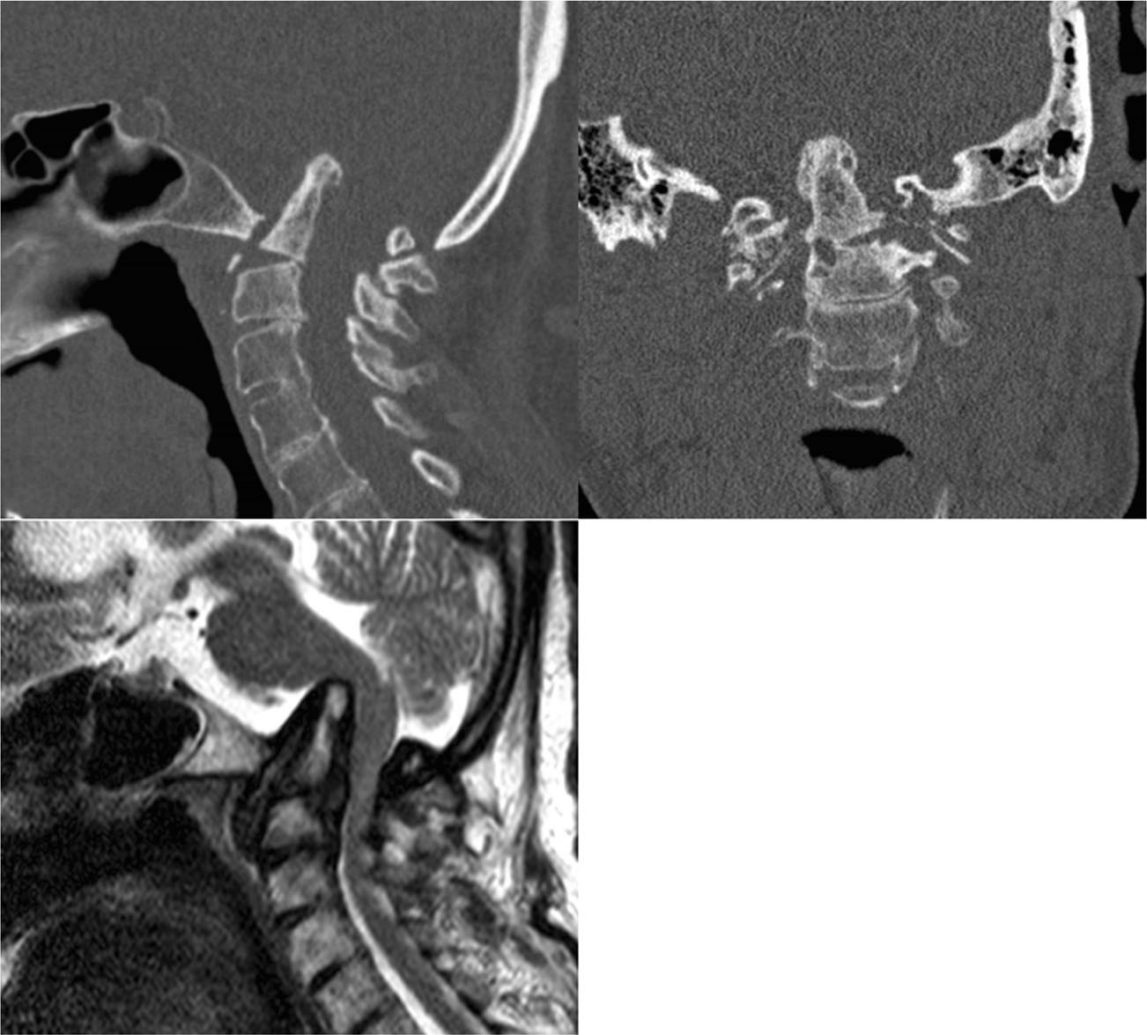
Basilar invagination in a 62-year-old female. Computed tomography, sagittal (**a**) and coronal (**b**) planes; magnetic resonance imaging, sagittal plane (**c**)

Time and again, hypotheses that had been approved as collective understandings of pathogenetic interactions are challenged and put into question. For the purposes of a thriving academia, it is equally as important to recognize these novel hypotheses as genuine avant-garde endeavors to further our scientific culture as it is mandatory to test and scrutinize them for scientific validity. In this nature, we may bear witness to a fundamental change in the way we perceive and understand the group of entities encompassing malformations of the craniovertebral junction (CVJ), mainly represented by the *Chiari malformation type I* (CM, Fig. 1) and *basilar invagination* (BI, Fig. 2). The academic community owes the recently sparked discourse on this intricate topic to Prof. Atul Goel, who has been pioneering his hypothesis that addresses the very principles of CVJ abnormalities and their genesis. Based on his longstanding and accredited experience on the surgical treatment of CVJ abnormalities in several original investigations, Goel was able to formulate an assumption that would entail a drastic change in the way these entities are treated surgically [30, 32, 33, 35, 37, 38]. His proposals have been met with both enthusiasm and dismissal, but no thorough review of his hypothesis against the available evidence has been conducted yet [8, 51, 53, 94]. In this paper, we examine publications supporting or opposing Goel’s hypothesis based on their respective clinical results.

### Definitions

Due to the manifold nomenclatures and criteria that govern the diagnosis of a CVJ malformation, it is crucial to settle definitions before proceeding with any discussion. The CVJ entities have traditionally been regarded as developmental anomalies that generally manifest in the pediatric population or in the middle stages of adulthood [38, 73]. The terminology has been compounded in the past and only crystallized with the increasing number of publications diverting their attention to this matter, while *basilar invagination* designates the primary, developmentally formed invasion of the odontoid process into the foramen magnum, *basilar impression* refers to a secondarily acquired protrusion due to softening of the skull, whereas *cranial settling* is specifically reserved for rheumatoid arthritis. In addition, both CM and BI are closely associated with syringomyelia and purportedly share common pathomechanisms involving compromised cerebrospinal fluid (CSF) dynamics at the CVJ, that remain improperly understood [3, 14, 25, 27, 46, 47]. Despite these uncertainties, surgical treatment has seen favorable results for both CM and BI with low complication rates in the last decades [1, 17, 22, 46, 60, 84, 97]. The surgical strategies principally aim at increasing the posterior fossa volume in CM by suboccipital decompression, relieving brain stem compression in BI by a ventral approach or suboccipital decompression, and an occipitocervical stabilization procedure when instability of the CVJ is evident [60, 66, 73]. Contrasting these principles, Goel seeks to define a common denominator for the complex of CVJ abnormalities in the form of “central atlantoaxial instability,” which is to be addressed universally by sole atlantoaxial stabilization without any decompression procedure [33, 37].

The definitions of atlantoaxial instability itself vary throughout literature as do the treatment guidelines [26, 45]. Commonly, lateral radiographs of the CVJ demonstrating an atlantodental interval of at least 3 mm in adults and 5 mm in children indicate instability, whereas newer investigations by computed tomography set the cut-off at 2 mm for adults [50, 59, 80, 81, 93]. This definition accounts for the most commonly encountered anterior displacement of atlantoaxial instability, while Fielding and Hawkins alternatively devised a classification with respect to the direction of the atlantoaxial dislocation [26]. The heterogeneity of definitions may equally be founded in the lack of a comprehensive assessment of a healthy control group, which only few morphometric studies have undertaken so far. Some distinguishing hallmarks were identified, although they tend to differ between studies [5, 75, 96].

In a most recent publication, Lacy et al. only consider anterior displacements of the atlas over the axis by more than 5 mm and posterior displacements unstable and in need of surgical treatment, whereas rotatory facetal subluxation and translation by less than 5 mm should be evaluated for conservative treatment or surgery in selected cases [63]. By the distinction between stable and unstable atlantoaxial dislocation, virtually, all authors differ fundamentally to Goel’s hypothesis, which seeks to predefine a universal instability even without any radiographic abnormality [33].

## Methods

Relevant literature for this narrative review was compiled by two independent reviewers, who conducted an online literature search on October 24, 2019. Our search strategy was applied to the following databases: PubMed/Medline and ISI Web of Science. These were queried for the following keywords and MeSH (medical subject headings) terms: “Chiari malformation,” “Chiari malformation type I,” “atlantoaxial instability,” “craniocervical decompression,” atlantoaxial fusion,” “posterior fossa decompression.” After finding a consensus on eligible articles by both reviewers, studies were included in this narrative review. Relevant articles identified via cross-referencing were also included, if they directly concerned the abovementioned group of entities in an investigative manner.

The principal items of interest extracted from the collected studies were the study types, patient numbers, surgical procedures, rates of complications, and symptomatic improvements as well as follow-up duration. Ultimately, each clinical study’s results were assessed for them supporting or objecting the hypothesis of atlantoaxial instability producing CVJ abnormalities. In addition, we first provided a cohesive outline of the numerous articles published by Goel, which cover this particular subject.

## Results

### Goel’s hypothesis

Goel introduced and developed explanations for the association of the primary CVJ abnormalities over the course of several publications. In his series of 190 surgically treated patients with BI, a clear methodological distinction was achieved to subdivide his cohort into group I without associated CM and group II with associated CM [38]. Mechanistically, he declared the dissociation of the odontoid from the anterior arch of the atlas to be pivotal for its upward migration and posterior angulation, thereby directly compromising the foramen magnum and compressing the brain stem. This presupposed instability of both the atlantoaxial and atlantooccipital complexes in group I patients. Group II patients in contrast exhibited upward migration of atlas, axis, and clivus *in unison*, which primarily produced a reduction of the posterior fossa volume and downward herniation of the cerebellar tonsils [38]. On this premise, selection of an appropriate surgical strategy incorporating ventral decompression, posterior decompression, posterior stabilization of the CVJ, or a combination of these would be feasible.

It is important to note that Goel manufactured the distinction on the basis of CM’s presence exclusively and deduced the aspect of facetal joint instability post hoc [29]. He renamed the labels to group A and group B in 2009, now prioritizing the observation of facetal instability in group A [29]. His focus on facetal orientation with subsequent instability of the naturally very mobile atlantoaxial joint as a pivotal factor deviated from the conventional view of atlantodental instability as a primary initiator. In Goel’s universal CVJ model, syringomyelia fits as a *tertiary response* to the reduced posterior fossa volume in CM (group B) patients [39]. He established a logical sequence of craniovertebral instability leading to atlantoaxial dislocation and thus the complex of CM, BI, and syringomyelia in 2005, when he first applied his philosophy in 12 patients undergoing C1-2 fixation without any form of decompression [44].

In 2009, Goel started to critically review the then current treatment paradigms for CVJ [29]. Based on his own observations, he began to differentiate the instability of group A patients from a primarily congenital pathogenesis without evidence of instability of the CVJ in group B patients. His hypothesis was underpinned by a craniometric study of 170 patients, the majority of which was conducted retrospectively. Treating patients with the unstable form of BI (group A) only by intraoperative atlantoaxial facetal distraction and stabilization resulted in an increase of craniovertebral height, neck length, and cervical lordosis in over 90% of individuals [43]. Goel thus argues that these pathomorphological hallmarks, which have been described numerous times since Klippel and Feil in 1912 and Chamberlain in 1939, are all subject to reversal, once the primary initiating neural compression has been alleviated [12, 23]. Several musculoskeletal changes, such as a short neck, assimilation of the atlas, neutralization of the craniospinal angulation, and a reduced cervical motion range, are thus merely natural adaptations [29, 34].

By extension, the development of CM with downward herniation of the tonsils would serve as a protective, sacrificial measure to mitigate compression on the brain stem, hence coining the term *Nature’s protective air bag* in his 2014 and 2015 publications [31, 33]. In his most controversially discussed feature, a portion of Goel’s cohort of patients did not exhibit any apparent preoperative atlantoaxial instability evident by facetal slippage on dynamic radiographs, but still underwent C1-2 stabilization. His assumption on this *central instability* or *type III atlantoaxial dislocation* being the driving pathogenetic factor was then validated intraoperatively by manual manipulation [33, 35]. Goel had, by then, uniformly resorted to C1-2 stabilization as a solution to both group A unstable BI and group B stable (*fixed*) BI with or without CM, abandoning any form of foramen magnum decompression. Ultimately, he deems any decompression procedure to be *akin to deflating the air bag*, which would only serve a temporary clinical effect and may be detrimental in the long run [33, 53]. In numerous further clinical and radiological studies, Goel consolidated his hypothesis mainly by reporting favorable clinical and electrophysiological recovery for adult and pediatric cohorts, albeit without offering substantial radiographic evidence for changes of syringomyelia and without methodologically tailored control groups to his cohorts [37, 41, 42, 86]. The resolution of suboccipital headaches, neck pain, and muscle spasms, even in the absence of manifest neural compression, inevitably validates the proposed pathomechanisms, he argues [36].

A thus far neglected element of Goel’s proposal lies in the long-term implications of craniocervical fusion in a generally adolescent population, which may affect the sagittal balance of the subaxial spine, height growth, and quality of life of children. Kennedy et al. report on favorable outcomes after craniocervical fusion in children, although a proportion of the cohort may experience difficulties in compensating for the fused segments [56]. The patient-reported outcomes seem to be satisfying, however, although no study has examined a homogenous CM population undergoing C1-2 fixation only [49, 79, 91].

### Basilar invagination—current treatment paradigm

The evolution of BI treatment has seen considerable advances within the recent decades, although not without controversies [15, 19, 20, 38, 55, 60, 61, 71, 73, 74, 85]. While it is equivocally agreed upon that incidental findings warrant conservative management, a consensus on a treatment algorithm for a progressive clinical deterioration has yet to be established, which may be owed to the diversity of CVJ abnormalities and their mutual interactions [58, 92].

The pathomechanisms that need to be addressed surgically are found in the instability, which manifests itself in a reducible BI, and the neural compression that may be directed from anterior or posterior. In 1980, Menezes and co-workers proposed a treatment algorithm tailored to these mechanisms, employing reduction and occipitocervical stabilization as a first measure for reducible BI and decompression from either anterior or posterior for irreducible BI [74]. In those cases with evidence of postoperative instability after decompression, a fusion procedure was carried out of variable extent via bone graft. The surgical techniques were refined consequently, with Goel often opting for C1-2 stabilization with lateral mass screws and rods in 1998, in addition to suboccipital craniectomy with duraplasty for associated CM and transoral decompression for irreducible BI [38].

Klekamp et al. reported their series of 323 patients with CM and BI undergoing treatment according to a refined algorithm [60]. Essentially, the algorithm intends for posterior fossa decompression (PFD) alone only when there are no signs of CVJ instability, ventral compression of the brain stem, and segmentation anomalies, regardless of the presence of BI. In any other case, a posterior stabilization, which extended from the occiput to C2, was added to the PFD. A transoral decompression was reserved for those patients presenting with caudal cranial nerve deficits caused by ventral compression. Klekamp’s rationale to primarily conduct a posterior stabilization for patients with assimilation of the atlas but a *stable* BI was to preemptively address postoperative instability, which he and others have observed in this specific subgroup. He argued that decompression alone eventually leads to musculoskeletal decompensation and debilitating chronic neck pain syndromes when no adjunct stabilization is performed simultaneously [60].

The principal decision-making has not been altered much since, although technical modifications to the anterior approaches continue to be investigated [21, 95].

### Chiari malformation type I—definitions

CM is defined as a descent of the cerebellar tonsil of at least 5 mm into the upper cervical canal. Several pathogenic mechanisms have been proposed. They can be divided mainly into (1) a volumetric disproportion between the posterior fossa and its contents (reduced embryologic development of the skull base), (2) hemodynamic/cerebrospinal fluid dynamic alterations resulting in increased intracranial pressure, (3) mass effect within the posterior fossa (e.g., tumors), and (4) low intraspinal pressure due to craniospinal intrathecal pressure imbalance (e.g., lumbo-peritoneal shunts) [9]. A detailed understanding of the underlying mechanism of the cerebellar tonsil descent is crucial for managing this cohort [67].

Noteworthy, CM may be associated with CVJ deformities. The simultaneous presence of craniocervical instability and the need for a proper preoperative work-up has been widely accepted. Initially, instability was mainly noted after suboccipital decompression and laminectomy [2]. Over time, failure of conventional surgical management has been attributed to coexisting craniocervical anomalies requiring reduction and OCF procedures [6]. In 2011, Tubbs and colleagues shared their surgical experience of 500 cases with pediatric CM over 2 decades. They identified several deformities in their cohort: 18% presented with spinal anomalies including scoliosis, 24% had a retroversion of the odontoid process, 3% a Klippel-Feil anomaly, and 8% an atlantooccipital fusion [90]. Logically, it is important to recognize subluxation, as failure to do so may result in clinical deterioration after decompressive surgery for CM, especially when laminectomy of the atlas is also performed [13]. The reported association of CM-I and atlantoaxial subluxation is around 30% [13, 65, 76]. Further another group tried to identify risk factors for the requirement of additional OCF in pediatric CM patients. In their retrospective review, concomitant BI and a clivoaxial angle below 125° have been identified to be associated with the need of OCF [6].

## Discussion

### Basilar invagination—discussion of evidence

It is essential to understand that Goel proposes a definitive uniform solution for a heterogeneous spectrum of CVJ abnormalities. By identifying atlantoaxial instability as a common denominator for CM, BI, atlantoaxial dislocation, and syringomyelia, it is possible to employ a single tried and tested technique as the entire management. To our knowledge, there is only one study that strictly reproduced Goel’s strategy of reduction and C1-2 stabilization without any decompression (Table 1) [83]. Most authors naturally undertake reduction of a dislocation whenever possible, in addition to stabilization in the reduced position and optional decompression measures when there is brain stem compression not amenable by reduction. Fusion usually extends to the occiput.

**Table 1:** Overview of clinical studies reporting outcome after fusion and decompression for BI

Authors	Year	Article type (level of evidence)	Population	Intervention	Outcome and conclusion
Brockmeyer	2011	Review & retrospective series (III)	*n* = 210; CM-I: 173 patients, CM-1,5: 37, scoliosis: 39, syrinx: 88, odontoid retroflexion: 43	PFD + C1 laminectomy ± duraplasty: 210, OCF 21, TO 10; 173 with CM-decompression ± duraplasty only; CM 1.5- > 21 OCF	Complex Chiari, “Chiari 1.5” and odontoid retroflexion > 5 mm benefit from TO and/or OCF
Chatterjee et al.	2019	Retrospective series (IV)	*n* = 9 adults; BI + CM + syrinx	Circumferential decompression (TO + PFD) + OCF	Neurological improvement in 88.9% of BI + CM, no radiographic follow-up
de Oliveira Sousa et al.	2017	Meta-analysis (III)	27 studies; *n* = 1451 adults; BI +/- CM	PFD +/- fusion	Clinical improvement 75–85% for different decompression techniques; no stratification by fusion
Dickman et al.	2012	Case report (IV)	16 years old male (Marfan) with BI, CM, syrinx	Reduction + staged TO + OCF	Fusion of a reduced BI improves CM and syringomyelia
Fenoy et al.	2008	Retrospective series (IV)	*n* = 234 children + adults; CM, BI, syrinx, CVJ abnormalities	Reduction + OCF +/- PFD +/- TO	Clinical improvement 92.0%, reduction and OCF for all unstable CVJ abnormalities (96%) +/- PFD (50.9%) +/- TO (44.0%)
Joaquim et al.	2014	Retrospective series (IV)	*n* = 26 adolescents and adults; BI + CM +/- syrinx	PFD +/- OCF /w reduction	Clinical improvement for most, not clearly defined; PFD for all BI +/- OCF for unstable BI in 10 cases
Kim et al.	2011	Case report (IV)	*n* = 2; BI	Reduction + C1-2/C1-4 + PFD	Clinical improvement in both, radiographic improvement
Klekamp	2015	Retrospective series (IV)	*n* = 323 mainly adults; CM +/- BI (14.2%)	PFD +/- OCF +/- TO	Clinical improvement 81.6%; fusion only for BI accompanied by ventral compression or segmentation anomaly
Menezes et al.	1980	Retrospective series (IV)	*n* = 17 children and adults; BI, CM, and other CVJ abnormalities mixed	Traction, PFD, OCF, C1-2, TO mixed	Clinical improvement 100%; primary aim was reduction and stabilization, irreducible pathologies need decompression
Ridder et al.	2015	Case report/clinical article (IV)	*n* = 1, 12 years old; BI + CM	PFD w/ tonsillar resection + OCF	No reduction; secondary brain stem compression 5 months postoperatively
Salunke et al.	2019	Retrospective series (IV)	*n* = 38 adults; BI + CM + AAD + syrinx	Preop. traction + C1-2	Clinical + radiographic improvement 91.9%; atlantoaxial fusion reduces ventral dural kinking, instability not primary problem
Scholtes et al.	2011	Case report (IV)	*n* = 1, 54 years old; BI + CM /w brain stem compression	Transnasal decompression	Almost full neurological recovery at 9 months postop., transnasal dens resection feasible for decompression without posterior fusion
Shuhui et al.	2016	Retrospective series (IV)	*n* = 43 adults; BI + CM + syrinx	Intraop. reduction + OCF + PFD + insertion of bone graft (iliac crest)	Clinical improvement 90.0%, syrinx regression in 80.0%
Wang et al.	2016	Retrospective series (IV)	*n* = 71 adults; BI + AAD + syrinx	Intraop. reduction + OCF + PFD	Clinical improvement 90.1%, successful BI reduction in 93.0%, syrinx reduction in 93.0%; posterior reduction and fusion improve CSF flow in reducible BI with ventral compression, PFD supplementary
Zileli and Cagli	2002	Retrospective series (IV)	*n* = 9 adults; BI + CM + syrinx	Circumferential decompression (TO + PFD) + OCF	Neurological improvement in 88.9% of BI + CM, no radiographic follow-up

*AAD*, atlantoaxial dislocation; *BI*, basilar invagination; *CM*, Chiari malformation type I; *C1-2*, atlantoaxial fusion; *OCF*, occipitocervical fusion; *PFD*, posterior fossa decompression; *TO*, transoral decompression

This strategy has been applied consistently by Menezes and Van Gilder in 1980 with significant functional improvement in each of their 17 cases [74]. Although the cohort was fairly heterogeneous at baseline, including cases with rheumatoid arthritis and traumatic atlantoaxial dislocation, the authors were the first to conceive a structured treatment algorithm, tailored to the primary pathology. They prioritized reduction of any atlantoaxial dislocation and its stabilization, while decompression measures were secondary for irreducible pathologies [74]. In a later study with pediatric patients suffering from osteogenesis imperfecta, their treatment algorithm was reapplied. Despite preoperative traction and successful occipitocervical fusion (OCF), BI progressed in 80% radiographically after completed management, although most of these were aged between 11 and 15 years and only 24% exhibited a recurrence of symptomatic brain stem compression [85]. In this publication, Menezes expressed the hypothesis of atlantoaxial instability being the progenitor of BI and other CVJ abnormalities, as he recognized that pediatric patients had a higher rate of reducible BI due to atlantoaxial instability than adult patients, where it had become *fixed* [70].

In the following years, several case reports and small series were published with strategies similar to Menezes’ [16, 48, 55, 57, 58, 68, 87]. The outcomes of all of these were reported to be excellent, although the populations were mainly pediatric. Goel et al. reported on an instantaneous benefit by traction for 82.0% of their BI patients without CM, while only 5% of those with CM improved immediately after traction [38].

Subsequently, a number of clinical studies reported outcomes after variable combinations of circumferential approaches with posterior reduction and fusion as well as posterior and anterior decompression, although the level of evidence is generally low (Table 1).

In a meta-analysis from 2017, different PFD techniques were compared across 27 pooled studies [17]. A postoperative improvement was noted in up to 85.0% of patients compared with a complication rate of 13.5% over 1451 patients with BI and/or CM. Details on radiological outcome or the rate of posterior fusions were not provided and only one study was reported as being randomized with methodological shortcomings, however.

In 2008, Fenoy and Menezes reported on 234 patients with CM and associated unstable CVJ abnormalities [24]. The authors comprised a catalog of instability criteria to screen their database of CVJ patients for instability and proceeded to fuse all of them from occiput to C2 or C3 with optional decompression measures. Again, postoperative improvement was noted in 92%. While some of the patients did not undergo any decompression procedure in addition to OCF, the authors do not provide exact reasoning for this decision-making.

In one of the most comprehensive series, Klekamp reported improvements in 81.6% of patients with CM and BI after treatment according to a predefined algorithm [60]. Among all patients, 14.2% had CM with BI and these were treated with sole PFD in 41.3% of cases compared with 95.3% in the CM without BI subgroup. The complication rate was significantly higher in the CM with BI group (35.6%) than in the CM without BI group (16.4%). Despite the higher proportion of fusion procedures in the CM with BI group, the radiographic reduction of syringomyelia occurred less often postoperatively [60]. In comparison, Goel and co-workers found a much higher incidence of BI with CM with 84.0%, and while also reporting an excellent improvement rate with 96.2% overall, only 6 out of 11 patients showed radiographic reduction of tonsillar herniation and syringomyelia [33]. In a later pediatric series, Goel and colleagues again used reduction and C1-2 instrumentation for CM with both unstable and *fixed* BI [40]. The authors report successful and sustained reduction in all patients, concomitant with marked clinical improvement.

The reason for postoperative improvement should thus be attributed in large part to the reduction, which indirectly decompresses the brain stem by a protruding odontoid, and partially in the stabilization, which serves to sustain this effect. This hypothesis was proposed by other authors [71, 83, 92]. Wang et al. analyzed the reduction of syrinx volume and CVJ alignment in 71 patients treated with OCF and bony suboccipital decompression after reduction. They found that for 93.0% of patients with satisfying reposition of the odontoid, a syrinx regression was achieved as well [92]. In a recent study, Salunke and colleagues reduced and fused 38 patients with CM and BI, of which 91.9% maintained both clinical and radiographical improvement after 6 months [83]. The authors did not conduct any craniectomy for decompression, as they conclude that the downward traction and repositioning of the odontoid suffices for indirect compression of the brain stem by neutralizing kinking of the dura. This supposedly services reestablishment of the CSF flow through the foramen magnum and enables reversal of the tonsillar herniation, which was observed in their study. Interestingly, all patients with iatrogenic dural laceration showed transient clinical worsening [83]. A PFD would likewise increase the posterior fossa volume and make space for the protective air bag, but not address the inherent pathogenetic mechanism, they argue. PFD therefore remains effective in treating the *sequelae* of CM with or without BI, albeit with a substantial risk for recurrence of CSF obstruction after decompression: in Klekamp’s study, the recurrence rates after PFD for CM without BI significantly increase with time and the number of surgeries performed, whereas those for CM with BI decrease with time and are mostly attributable to hardware failure [60].

Joaquim et al. applied a different surgical strategy for BI with concomitant CM in 2014. In their series of 26 patients, all but one underwent PFD primarily, while 9 patients with clear atlantoaxial instability on preoperative flexion radiographs received OCF as well [52]. Most of the patients improved clinically after an average follow-up of 9 months, although no radiographic follow-up was reported to evaluate the development of tonsillar herniation and odontoid migration.

It should be noted that, while Goel intentionally limited his instrumentation to C1-2, more than half of his reported patients exhibited an assimilated atlas, which biomechanically rendered his C1-2 approach an OCF in many cases [33]. As pointed out by Jea and Goel himself, exposing the joints and screw insertion sites of the atlas may impose substantial risk to the vertebral arteries and extradural venous plexuses of the CVJ, which appears wholly unjustified when considering the possibility of an OCF in case of an already existing atlas assimilation instead [33, 42, 51].

The aforementioned observations emphasize the need to critically reconsider abolishing a toolkit of surgical strategies in favor of a single one, especially when there may be absent preoperative evidence of the decisive atlantoaxial instability in a significant number of cases, i.e., a *Type III instability*. More so than any author so far, it is commendable that Goel strives for a more streamlined solution, which in turn makes for an uneven comparison of the outcomes between his and other series. Relying on a *one size fits all* procedure is certainly warranted for a subgroup of patients, for whom unstable BI represents the primary CVJ abnormality. The current state of evidence, however, does not allow for an unequivocal adoption of his novel hypothesis, even when his outcomes and expertise have produced compelling results. This argument is further emphasized by the excellent outcomes and complication rates of established treatment strategies.

A comparison of surgical strategies in a representable patient cohort under controlled circumstances is necessary, since the delicate mechanistic explanations are difficult to prove without studies employing homogeneous methodologies and focuses.

### Chiari malformation type I—discussion of evidence

As mentioned above, several processes can lead to or are coexisting with CM. Hence, a detailed understanding is necessary to provide a case-based approach. Over time, several treatment options have been introduced. As pointed out by Brockmeyer, it is important to recognize “complex” Chiari cases as they may require additional surgical interventions aside from a typical suboccipital decompression [7].

PFD with or without duraplasty (PFDD) has been and is still widely used. To date, the largest meta-analysis dealing with PFD +/- PFDD by Chai et al. demonstrated that both procedures are effective options in most cases [11]. PFDD is associated with a larger reduction in syrinx size, but with a higher incidence of CSF leakage and aseptic meningitis. Despite these favorable results, it has to be mentioned that resolution of syringomyelia has been shown after posterior C1-C2 distraction and fusion as well [82].

Over time, it has been increasingly recognized that patients with CM and concomitant CVJ abnormalities are more likely to deteriorate clinically after PFD [13, 72]. This may be especially true for patients receiving PFD plus C1 laminectomy with (undiagnosed) AAD. In a single center retrospective analysis on patients with pediatric CM types 1 and 1.5 by Brockmeyer, 210 patients were identified who received surgery. PFD with C1 laminectomy was performed in all cases and OCF was needed in around 10% of all patients. Notably, none of the CM type I patients required OCF or odontoid resection, but more than half of patients with the more complex CM type 1.5 underwent OCF and more than 20% received additional odontoid resection [7]. Still, the PFD technique represents an established surgical procedure adopted and pursued by the majority of specialists involved with the treatment of CM. Several studies on long-term outcome provide testament to excellent results. Reported success rates reach 84% even after 5 years of follow-up as well as negligible surgical morbidity and mortality [28, 62, 88, 89]. These results have to be opposed with the outcome of the novel C1-2 fixation strategy, for which long-term outcome is still sparse [33].

In the aforementioned comparative study by Klekamp, the influence of concomitant BI in patients with CM pathology was clearly demonstrated [60]. Out of 323 CM-I patients, 46 (14.2%) also had BI. PFDD was performed in all cases without BI and OCF was added in only 4 patients due to instability. In patients with concomitant BI but no ventral cord compression, PFD was also conducted. The strategy changed in cases with ventral spinal cord compression, where transoral decompression followed by posterior decompression and fusion was conducted. Hence, he concluded that CM patients without BI or with BI but no significant ventral spinal cord compression can be managed by PFD alone. The issue of secondarily progressive instability after PFD is a particularly controversial topic without profound evidence. Again, reports on postoperative instability range widely between 9 and 95%, being relegated to retrospective case series and case reports with varying definitions [2, 4, 10, 64, 69]. Generally, most authors seem to be concerned predominantly about instability after anterior decompression of the odontoid and consistently report instability rates of 72% with significant neurological morbidity [60, 77, 78].

By Goel’s theory, all CM patients have subtle or radiologically apparent atlantoaxial instability, which is the hallmark of the pathophysiological cascade [33]. We fully agree that the coexistence of BI and AAD with CM should not be missed with the available data nowadays [13, 84]. However, previous studies clearly demonstrated clinical efficacy with decompression alone and probably remains a suitable treatment option for CM with no signs of instability [11, 54]. In summary, we agree with Deora and colleagues that in patients with pure CM with symmetrical C1/2 joints and no signs of instability, PFD and duraplasty is an established, effective, and safe treatment option with excellent long-term outcome [18, 28, 62, 89]. In patients with instability, C1-2 fusion with distraction or OCF with or without transoral decompression in selected cases are potential treatment options that have to be considered in this patient population.

## Conclusion

During recent years, a novel treatment strategy for CVJ abnormalities has been developed, with atlantoaxial instability being declared the abnormalities’ original cause. We conducted a narrative literature review of the evidence for this topic, which primarily stems from retrospective investigations and case reports. As current evidence does not clearly support or refute this hypothesis, prospective controlled studies incorporating functional and patient-focused outcome parameters are necessary before unequivocally adopting one single treatment strategy.
